# Calgary score and modified calgary score in the differential diagnosis between syncope and genetic generalized epilepsy in children

**DOI:** 10.1038/s41598-023-39338-5

**Published:** 2023-07-31

**Authors:** Mehmet Tolga Köle, Safiye Günes Sağer, Utku Batu, Nilüfer Çetiner Çine, Yakup Çağ, Yasemin Akin

**Affiliations:** 1 Department of Pediatrics, University of Health Science, Kartal Dr. Lutfi Kirdar City Hospital, Şemsi Denizer Cad. E-5 Karayolu Cevizli Mevkii, Kartal, 34890 Istanbul, Turkey; 2 Department of Pediatric Neurology, University of Health Science, Kartal Dr. Lutfi Kirdar City Hospital, Istanbul, Turkey; 3 Department of Pediatrics, Van Education and Research Hospital, Van, Turkey; 4 Department of Pediatric Cardiology, Koşuyolu Highly Specialized Training and Research Hospital, Istanbul, Turkey

**Keywords:** Epilepsy, Paediatric research

## Abstract

The purpose of the study is to explore the use of Calgary scoring (CS) and Modified Calgary scoring (MCS) in the differentiation of genetic generalized epilepsy and syncope in children. The study involved 117 patients aged < 18 years who presented to our hospital’s pediatric neurology outpatient clinic with TLOC between June 2020 and June 2022. In addition to CS and MCS scoring, all patients were subjected to statistical analysis based on their age, sex, number of episodes and distribution during the day, duration of syncope, and family history. Seventy-one patients with syncope and 46 with epilepsy were included in the study. At a CS value >  − 1, sensitivity was 86.9% and specificity 63.4%, while at an MCS value >  − 1, sensitivity was 76.1% and specificity 71.8%. CS had less specificity and sensitivity in predicting epilepsy when focal epilepsies were excluded. Abnormal behavior noted by bystanders, including witnessed unresponsive, unusual posturing, or limb jerking? (Q5) emerged as the most important question for the detection of epilepsy. Compared with other syncope findings, loss of consciousness during prolonged sitting or standing (Q9) emerged as the most important for the detection of syncope.

## Introduction

The etiology of transient loss of consciousness (TLOC) in childhood is difficult to determine, although the most common reported causes in childhood are syncope and epilepsy^1,2^. A previous study reported that approximately 40% of children (mostly girls) experience TLOC at least once in their lives^3^.

In their study of 4352 children, Hu et al. reported an incidence of syncope of 17.37%, and a mean age at experiencing syncope of 16 years in children^4^. However, the lifetime prevalence of epilepsy has been reported at 7.60 per 1000 individuals^5^. Notably, focal epilepsies have been found to be more common than generalized epilepsies (GE) in both adult and pediatric patient groups^6,7^.

The most common cause of TLOC in children is neural-mediated syncope (NMS) caused by cardiovascular mechanisms, which results in vasodilation and/or bradycardia^8^. Epilepsy is another cause of TLOC^9^. Sheldon et al. developed the Calgary scale (CS) that compares the symptoms and signs of epilepsy with syncope in order to facilitate the diagnostic process^10^. Zou et al. evaluated the CS in the pediatric population and reported its use in differentiating between syncope and epilepsy in children^8^. However, assessment of the questions used in the CS for evaluating epilepsy revealed that some were more valid for focal epilepsies than for genetic generalized epilepsy (GGE). Is the CS useful for GGE? Or how often are findings suggestive of syncope, such as dizziness, palpitations, and nausea, seen in patients with GGE? The purpose of this study was aimed to seek answers to these questions and to explore the use of CS and MCS in the differentiation of epilepsy and syncope in children.

## Materials and methods

### Study design and participants

The study involved 117 patients aged < 18 years who presented to the Istanbul Kartal Dr. Lütfi Kırdar City Hospital pediatric neurology outpatient clinic, Turkey, due to TLOC between June 2020 and June 2022. The study was approved by the hospital ethics committee (number 2022/514/232/4; date: 26 August 2022), and was conducted in accordance with the principles of the Declaration of Helsinki. Informed consent was obtained from parents or legal guardian(s) of patients. The study was conducted retrospectively. Patients who presented to our clinics with TLOC underwent neurological examinations, electroencephalogram (EEG) monitoring, and were evaluated by Pediatric Cardiology physicians. Subsequently, the CS and MCS were administered to the patients by two physicians.

According to the literature, detailed anamnesis and physical examination are sufficient to differentiate reflex syncope and epilepsy in children. We also used this algorithm in differential diagnosis in our study^11,12^. The diagnosis of syncope was made in patients with normal EEG by using a detailed history, physical examination and 12-lead electrocardiogram in the first stage in accordance with the recommendations of the Canadian Cardiovascular Society^11^. Patients diagnosed with GGE according to the International League Against Epilepsy 2017 classification were included in the study^13^. Among the patients with normal EEG results, at least three EEG examinations were performed within one year. The diagnosis of GGE was made on clinical grounds supported by the presence of typical interictal EEG discharges (generalized spike-wave activity on EEG) and medical history^13^.

In addition to the CS and MCS scoring, all patients were subjected to statistical based on their age, sex, family history, number of episodes, distribution of episodes during the day, syncope duration, and family history. Patients with focal epilepsy, intellectual disability (intelligence quotient, 70), dysmorphic findings on the face or body, structural lesions at cranial MRI, chronic diseases capable of causing comorbidities other than GGE, and failing to provide written consent were excluded from the study. Also, only patients who applied due to TLOC were included in the study. Patients who could not provide a detailed medical history through their relatives and patients who described seizures were not included.

### Cardiac evaluation

All patients were evaluated in terms of etiology of syncope in the pediatric cardiology department, based on detailed history, physical examination, electrocardiography, and transthoracic echocardiography. All patients were also examined in terms of arterial blood pressure and pulse while lying down, sitting up, and finally standing for 10 min. Changes in heart rate and blood pressure values were recorded.

Orthostatic hypotension was defined as a 20 mmHg decrease in systolic blood pressure and a 10 mmHg decrease in diastolic blood pressure within 5 min of standing up from the supine position on the examination table^14^. Postural orthostatic tachycardia syndrome was defined as an increase in heart rate of > 30 beats per minute (bpm) or a heart rate of > 120 bpm within the first 10 min after standing up^14^. Vasovagal syncope was diagnosed in line with the definition in the 2018 European Society of Cardiology guidelines^15^.

### EEG recordings

The electrodes were placed in line with the international 10–20 system. All patients underwent 60-min sleep and wake EEG monitoring including hyperventilation, with sleep deprivation and photic stimulation.

### Calgary and modified calgary scores

The CS consists of nine ‘yes or no’ questions about medical history, triggering factors, and conditions and symptoms of TLOC^8^. Based on the responses to the questions, scores are calculated depending on the increase in the risk of epilepsy. In contrast to the CS, the third question on the MCS investigates whether the loss of consciousness occurred during sleep, rather than emotional stress^8^. Furthermore, the seventh question of the scales inquires about presyncopal symptoms such as dizziness, palpitations, or nausea.

### Statistical analysis

The study data were evaluated using IBM SPSS Statistics Standard Concurrent User version 26 (IBM Corp., Armonk, New York, USA) and MedCalc® Statistical Software version 19.6 (MedCalc Software Ltd., Ostend, Belgium). Descriptive statistical values were presented as number of units (*n*), percentage (%), mean ± standard deviation, median, minimum, maximum, and interquartile range. Normality of distribution of numerical variables was evaluated using the Shapiro–Wilk test. Homogeneity of variances was evaluated using Levene’s test. Intergroup comparisons of numerical variables were performed using the independent samples t-test when the data were normally distributed and the Mann–Whitney Utest in case of non-normal distribution. The chi-square test was used to compare categorical variables. The performances of CS and MCS in predicting epilepsy were evaluated using receiver operating characteristic curve analysis. The difference in the presyncope distribution of 50% within the groups was assessed using the one-sample binomial test. A *p*-value of < 0.05 was considered statistically significant.

### Institutional review board approval

Approval for the study was granted by the Kartal Dr. Lütfi KIRDAR City Hospital Clinical Trials Ethics Committee (no. 2022/514/232/4 dated 26 August, 2022), and informed consent was obtained from parents or legal guardian(s) of patients.

## Results

### General characteristics.

One hundred seventeen patients, 71 with syncope and 46 with epilepsy, were included in the study. Fifty (70.4%) patients in the syncope group and 27 (58.7%) in the epilepsy group were women (Table 1). Sex distributions in the two groups were similar. The patients’ ages ranged from 2.58 to 17.92 years, and there was no significant age difference between the two groups. The CS and MCS values of patients with epilepsy were statistically higher than the values of those with syncope (Table 1). Notably, the cardiac distributions of the two groups were statistically similar. Thirteen (18.3%) and nine (19.6%) patients in the syncope and epilepsy groups, respectively, experienced > 10 TLOC repetitions. Numbers of recurrences were similarly distributed between the groups. Family history, dizziness, and headache distributions were also similar between the two groups. However, maximum TLOC time was higher among patients with epilepsy. Thirty-nine (54.9%) patients from the syncope group and 29 (63.0%) from the epilepsy group reported that fainting could occur at any time of the day. The distributions of fainting times during the day were similar in both groups.

**Table 1:** A comparison of descriptive characteristics.

	Groups		Test statistics	
	Syncope *n* = 71	Epilepsy *n* = 46	Test value	*p*-value
Sex, *n* (%)				
Female	50 (70.4)	27 (58.7)	1.706^†^	0.233
Male	21 (29.6)	19 (41.3)		
Age, (*years*)				
*Mean* ± *SD*	13.28 ± 3.35	12.83 ± 3.57	0.694^&^	0.489
*Min–Max*	3.08–17.75	2.58–17.92		
Calgary score				
*M* (*IQR*)	− 1.0 (2.0)	1.0(2.0)	5.264^‡^	** < 0.001**
Modified calgary score				
*M* (*IQR*)	− 2.0 (3.0)	1.0 (2.2)	5.203^‡^	** < 0.001**
Cardiac, *n* (%)	*n* = 27	*n* = 9	3.021^†^	0.443
Bicuspid aorta	1 (3.7)	0 (0.0)		
Mitral regurgitation	0 (0.0)	1 (11.1)		
Normal	26 (96.3)	8 (88.9)		
Number of repetitions, *n* (%)				
1–5	51 (71.8)	34 (73.9)	0.396^†^	0.902
5–10	7 (9.9)	3 (6.5)		
> 10	13 (18.3)	9 (19.6)		
Family history,*n* (%)				
No	25 (35.2)	10 (21.7)	2.417^†^	0.150
Yes	46 (64.8)	36 (78.3)		
Dizziness, *n* (%)				
No	35 (49.3)	28 (60.9)		
Yes	36 (50.7)	18 (39.1)	1.505^†^	0.257
Maximum syncope time (min)				
*M* (*IQR*)	1.0 (2.0)	2.0 (4.0)	2.704^‡^	**0.007**
Fainting time, *n* (%)				
Any time	39 (54.9)	29 (63.0)	2.182^†^	0.530
Morning	24 (33.8)	12 (26.1)		
Midday	1 (1.4)	2 (4.3)		
Evening	7 (9.9)	3 (6.5)		

Significance values are in bold.

*SD* Standard deviation, *M* Median, *IQR* Interquartile range, ^†^: Chi-square test, ^&^: Independent sample t-test, ^‡^: Mann–Whitney U test.

### Calgary and modified calgary scores

The CS consists of nine questions. The first six questions predict epilepsy, and the following three predict syncope. In the present study, the CS was applied to children based on their clinical characteristics. Q3 on the CS inquires whether “loss of consciousness occurs with emotional stress,” whereas in the MCS, Q3 inquires whether “loss of consciousness occurs during sleep.” In the present study, Q5 and Q6 emerged as statistically significant for identifying epilepsy (*p* < 0.001; *p*: 0.009), and Q7 and Q9 as significant for identifying syncope (*p*: 0.004; *p*: 0.002) (Table 2). No significant difference was observed between the two groups in terms of Q1, Q2, Q3, Q3', Q4, or Q8 (*p* > 0.05) (Table 2).

**Table 2:** A comparison of the calgary and modified calgary scale responses between the epilepsy and syncope groups.

	Groups		Test statistics	
	Syncope *n* = 71	Epilepsy *n* = 46	Test value	*p*-value
Q1. Waking up with a cut tongue?			0.106^†^	> 0.999
No	67 (94.4)	44 (95.7)		
Yes	4 (5.6)	2 (4.3)		
Q2. Prodromal deja vu or jamais vu?			0.031^†^	> 0.999
No	53 (74.6)	35 (76.1)		
Yes	18 (25.4)	11 (23.9)		
Q3. Loss of consciousness with emotional stress?			0.613^†^	0.452
No	38 (53.5)	28 (60.9)		
Yes	33 (46.5)	18 (39.1)		
Q3’. Loss of consciousness during sleep?			0.488^†^	0.570
No	37 (52.1)	27 (58.7)		
Yes	34 (47.9)	19 (41.3)		
Q4. Head turning to one side during loss of consciousness?			3.218^†^	0.110
No	69 (97.2)	41 (89.1)		
Yes	2 (2.8)	5 (10.9)		
Q5. Abnormal behavior noted by bystanders, including witnessed unresponsive, unusual posturing, or limb jerking?			13.757^†^	** < 0.001**
No	48 (67.6)	15 (32.6)		
Yes	23 (32.4)	31 (67.4)		
Q6. Postictal confusion?			7.069^†^	**0.009**
No	41 (57.7)	15 (32.6)		
Yes	30 (42.3)	31 (67.4)		
Q7. Any presyncope, such as dizziness, palpitation, or nausea?			8.701^†^	**0.004**
No	25 (35.2)	29 (63.0)		
Yes	46 (64.8)	17 (37.0)		
Q8. Diaphoresis before a spell?			3.286^†^	0.098
No	58 (81.7)	43 (93.5)		
Yes	13 (18.3)	3 (6.5)		
Q9. Loss of consciousness with prolonged sitting or standing?			10.516^†^	**0.002**
No	36 (50.7)	37 (80.4)		
Yes	35 (49.3)	9 (19.6)		

Significance values are in bold.

^†^Chi-square test.

**Figure 1:**
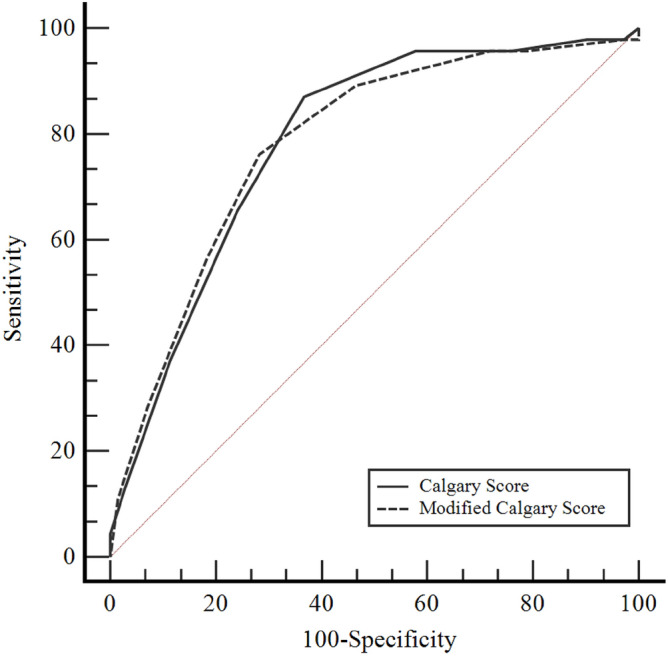
Comparison of receiver operating characteristic curves for the calgary and modified calgary scores.

According to the findings in Table 3, the area under the curve (AUC) values for CS and MCS were 0.786 and 0.782, respectively (Fig. 1) (Table 3). The AUC values for both scores were statistically significant. The optimum cut-off value for both scores was >  − 1. At a CS value of >  − 1, sensitivity was 86.9% and specificity 63.4%. At an MCS value of >  − 1, sensitivity was 76.1% and specificity 71.8% (Table 4). Although CS has higher sensitivity than MCS in identifying patients with true epilepsy, MCS has higher specificity than CS in identifying true normal individuals.

**Table 3:** Receiver operating characteristic curve (ROC) analyses for the calgary and modified calgary scores in predicting epilepsy.

	*AUC* (95% *CI*)	*p*-value	Cut-off point	Sensitivity (95% *CI*)	Specificity (95% *CI*)
CS	0.786 (0.700–0.856)	< 0.001	> − 1	86.9 (73.7–95.1)	63.4 (51.1–74.5)
MCS	0.782 (0.697–0.853)	< 0.001	> − 1	76.1 (61.2–87.4)	71.8 (59.9–81.9)

*AUC* Area under the curve, CI Confidence interval.

**Table 4:** Sensitivity and specificity values of the calgary and modified calgary scales for all scores.

Criterion	Calgary score				Modified calgary score			
	Sensitivity	95% *CI*	Specificity	95% *CI*	Sensitivity	95% *CI*	Specificity	95% *CI*
> − 6					97.8	88.5–99.9	0.0	0.0–5.1
> − 5	97.8	88.5–99.9	2.8	0.3–9.8	97.8	88.5–99.9	2.8	0.3–9.8
> − 4	97.8	88.5–99.9	9.9	4.1–19.3	95.7	85.2–99.5	21.1	12.3–32.4
> − 3	95.7	85.2–99.5	23.9	14.6–35.5	95.7	85.2–99.5	28.2	18.1–40.1
> − 2	95.7	85.2–99.5	42.3	30.6–54.6	89.1	76.4–96.4	53.5	41.3–65.5
> − 1	87.0	73.7–95.1	63.4	51.1–74.5	76.1	61.2–87.4	71.8	59.9–81.9
> 0	65.2	49.8–78.6	76.1	64.5–85.4	56.5	41.1–71.1	81.7	70.7–89.9
> 1	37.0	23.2–52.5	88.7	79.0–95.0	28.3	16.0–43.5	93.0	84.3–97.7
> 2	13.0	4.9–26.3	97.2	90.2–99.7	10.9	3.6–23.6	98.6	92.4–100.0
> 3	4.4	0.5–14.8	100.0	94.9–100.0	0.0	0.0–7.7	100.0	94.9–100.0
> 4	0.0	0.0–7.7	100.0	94.9–100.0				

*CI* Confidence Interval.

In Table 5, presyncope findings were present in 64.8% of the syncope group, differing significantly from 50% distribution. Presyncope findings were present in 37% of the patients in the epilepsy group, and the difference between the patients with and without this finding was not statistically significant.

**Table 5:** A Comparison of the presence of presyncope between the two groups according to 0.5 probability values.

	Syncope *n* = 71	Epilepsy *n* = 46
Any presyncope, such as dizziness, palpitation, or nausea? *n* (%)		
No	25 (35.2)	29 (63.0)
Yes	46 (64.8)	17 (37.0)
Test statistics	*z* = 2.374; *p* = **0.018**	*z* = 1.622; *p* = 0.105

Significance values are in bold.

*z*: One-sample Binomial test.

## Discussion

TLOC is a common symptom of both epilepsy and syncope in childhood, and differential diagnosis is difficult^16^ since the prevalence of syncope and juvenile epilepsy is highest at 5–18 years^4,17^. Additionally, emotional stress increases with adolescence^18^. Previous studies have reported epilepsy being diagnosed instead of vasovagal syncope in the pediatric group, or that a significant proportion of patients followed-up for epilepsy actually had syncope^19,20^. The purpose of the present study was to demonstrate the use of the CS and MCS, developed to assist with the differential diagnosis of children with GGE and syncope.

In a study involving 201 pediatric cases, Zou et al. reported that the CS and MCS can be used in the detection of epilepsy in children^8^. In that study, the sensitivity and specificity of the CS in differentiating syncope and epilepsy were 91.46% and 95.80%, respectively, compared to 92.68% and 96.64% for the MCS^8^.

The question still arises of whether the CS can still be used in this distinction if only juvenile-onset GGE were to be considered. The CS and MCS were therefore used in both groups in this study. Cases of epilepsy with normal EEG have been reported in the literature^9^, as well as patients with cardiac TLOC who have received anti-epileptic therapy for many years^19^. Sheldon et al. reported that the CS accurately classifies 94% of patients and diagnoses convulsions with 94% sensitivity and 94% specificity^10^. In contrast to those results, sensitivity and specificity in the present study were 86.9% and 63.4% for the CS and 76.1% and 71.8% for the MCS, respectively. The difference between our study and Zou et al. is that focal epilepsy was excluded from the present research. Therefore, when considering GGE in childhood, the presence of syncope-related findings or CS calculation does not seem to be sufficient for diagnosis. To the best of our knowledge, this is the first study on the topic in the pediatric age group.

Zou et al. described questions Q1, Q4 and Q5 as significantly reliable in the detection of epilepsy^8^. In addition, all types of epilepsy, including generalized and focal, were included in their study. In contrast to previous research, Q5 emerged as the most reliable question in detecting epilepsy in the current study. However, Q1 and Q4 were not significantly reliable in the syncope and epilepsy groups.

In the CS, presyncope findings are included in a single question, Q7—“Any presyncope, such as dizziness, palpitation, or nausea”^8^. Notably, Q7 was found to be significantly highly reliable in the syncope group compared to the epilepsy group and can also be used in the differential diagnosis of epilepsy. However, we assessed dizziness separately from the other two parameters and observed no significant difference between the syncope and epilepsy groups. Dizziness was considered a finding of presyncope, the prevalence of which was also relatively high in the GGE. Also, Q6 was found to be significantly reliable for differentiating epilepsy and syncope. Comparison of the presence of presyncope in the groups according to 50% probability values revealed that presyncope findings were significantly higher in the syncope group, although no significant difference was found in the epilepsy group. The presence of presyncope findings alone should not therefore be used to exclude GGE in the pediatric age group. The finding that most supports the presence of syncope in this study was Q9. No statistically significant difference was determined between the two groups in terms of presence of a family history of epilepsy.

The limitations of this study include its single-center nature, the number of participants was low, the absence of Video-EEG monitoring, and the fact that the tilt test could not be performed.

## Conclusions

The peak ages of patients in the pediatric age group with GGE and syncope were extremely similar. It is difficult to distinguish epilepsy from syncope in the presence of TLOC. Presyncope findings, such as dizziness, diaphoresis, and palpitations, are also frequently observed in GGE. However, waking up with a cut tongue and turning the head to one side during loss of consciousness, predictors of epilepsy on the CS, were not observed in GGE. CS had less specificity and sensitivity in predicting epilepsy when focal epilepsies were excluded. However, the CS can prevent unnecessary use of antiepileptic drugs in patients who cannot be diagnosed with epilepsy using repetitive EEGs. Abnormal behavior noted by bystanders, including witnessed unresponsive, unusual posturing, or limb jerking? (Q5) was found to be the most important question for the detection of epilepsy. Compared with other syncope findings, loss of consciousness during prolonged sitting or standing (Q9) emerged as the most important question for the detection of syncope.

## Data Availability

The data that support the findings of this study are available on request from the corresponding author. The data are not publicly available due to privacy or ethical restrictions.
